# Effect of Sepatronium Bromide (YM-155) on DNA Double-Strand Breaks Repair in Cancer Cells

**DOI:** 10.3390/ijms21249431

**Published:** 2020-12-11

**Authors:** Dusana Majera, Martin Mistrik

**Affiliations:** Laboratory of Genome Integrity, Institute of Molecular and Translational Medicine, Faculty of Medicine and Dentistry, Palacky University, 779 00 Olomouc, Czech Republic; dusana.majera@upol.cz

**Keywords:** survivin, YM-155, DNA damage, molecular mechanism of action

## Abstract

Survivin, as an antiapoptotic protein often overexpressed in cancer cells, is a logical target for potential cancer treatment. By overexpressing survivin, cancer cells can avoid apoptotic cell death and often become resistant to treatments, representing a significant obstacle in modern oncology. A survivin suppressor, an imidazolium-based compound known as YM-155, is nowadays studied as an attractive anticancer agent. Although survivin suppression by YM-155 is evident, researchers started to report that YM-155 is also an inducer of DNA damage introducing yet another anticancer mechanism of this drug. Moreover, the concentrations of YM-155 for DNA damage induction seems to be far lower than those needed for survivin inhibition. Understanding the molecular mechanism of action of YM-155 is of vital importance for modern personalized medicine involving the selection of responsive patients and possible treatment combinations. This review focuses mainly on the documented effects of YM-155 on DNA damage signaling pathways. It summarizes up to date literature, and it outlines the molecular mechanism of YM-155 action in the context of the DNA damage field.

## 1. Introduction

Survivin, also known by the name BIRC5, is a multifaceted protein crucial for cell division and possesses a very appealing feature for use in clinical oncology, which is the inhibition of apoptosis [1,2]. Survivin is not the main focus of this review, but it was the initial target that led to the discovery of YM-155. For more detailed insights summarizing the knowledge of more than two decades of research on survivin’s biology, its functions in the regulation of apoptosis and cell division, and strategies for its targeting, see more dedicated reviews [3,4,5]. YM-155 was first introduced as the imidazolium-based survivin suppressant with potent antitumor activities in hormone-refractory prostate cancer (HRPC). The initial screen of the chemical compound library to identify a specific survivin inhibitor was conducted using a survivin promoter-luciferase reporter assay. The YM-155 treatment resulted not only in suppressed survivin expression, but also induced apoptosis. The potent antitumor activity was first reported for the HRPC xenografted mouse model, and the same mice displayed a higher distribution of YM-155 within the tumor mass than in plasma [6]. This study inspired extensive research in the following years, which unquestionably confirmed the efficacy of YM-155 alone or in combination with other drugs in various cultured cancer cell lines and xenografted mouse models involving many cancer types [7]. Shortly after, YM-155 went into phase I clinical trials against advanced solid malignancies or lymphoma showing promising preliminary antitumor activity. Importantly, YM-155 also seemed to be safe and well-tolerated [8]. Highly anticipated subsequent clinical trials, which are excellently reviewed in [7], yielded relatively modest results, despite the high anticancer potency of YM-155 in preclinical studies.

The anticancer potency of YM-155 was initially explained via survivin’s role in multiple cellular homeostasis pathways [9]. However, reports from upcoming years provided substantial evidence behind the drug’s potency and found that it might be due to its promiscuous nature as many other molecular targets of YM-155 were subsequently revealed [7]. Interestingly, in the early years of the drug characterization, the sensitization effect of YM-155 to γ-radiation was reported for non-small cell lung cancer (NSCLC) both in vitro and in vivo. Additionally, treatment with YM-155 was shown to delay the repair of radiation-induced double-strand breaks (DSBs), as documented by DSB molecular marker γH2AX-foci analysis [10]. The authors of this study suggested that this might be due to survivin’s previously unknown role in DSB repair regulation [11]. Another study further supported this hypothesis by reporting increased γH2AX foci and persistent DNA DSBs after survivin silencing and pointed at nuclear survivin’s interaction with some of the proteins involved in DSB’s repair machinery [12]. However, later research provided the first direct evidence that YM-155 is a DNA damaging agent. The suppression of survivin is somewhat a secondary event as the concentration needed for γH2AX foci formation is significantly lower than those required by survivin inhibition [13]. The discovery of DNA damaging properties of YM-155 also introduced the point where the cancer-relevant molecular target of YM-155 was critically called into question [14].

Given that various tumors have different vulnerabilities that could be exploited therapeutically, knowledge of the drug’s precise action is highly essential. It can help predict which patients could benefit from such a treatment. Moreover, monotherapy is often not sufficient, and a thriving drug combination should be based on in-depth knowledge of the drug’s cellular mechanisms. In recent years, ongoing research conducted on YM-155 and its mode of action yielded a more in-depth view of its impact on DNA damage response.

Altogether, this review aims to better understand the molecular mechanism of YM-155’s mode of action; since YM-155 still represents a possible drug to be used in cancer treatment, this is critical. The exact molecular target of YM-155 was and still is a matter of debate. Nevertheless, recent research is proving that YM-155 is mostly related to DNA damage signaling pathways, as discussed in the following.

## 2. Selected DNA Damage Response Pathways Relevant to the YM-155’s Mode of Action

Human cells have to deal with an estimated 70,000 lesions [15] with mutagenic potential on a daily basis. If unrepaired, DNA damage can result in genome instability, a prerequisite for malignant cells’ transformation. Since the genome is under constant diverse attacks from endogenous or exogenous sources, cells have evolved highly sophisticated machinery for sensing and repairing DNA damage [16]. At the same time, enhanced endogenous DNA damage and replication stress are typical hallmarks of cancer cells.

Ataxia-telangiectasia mutated (ATM) kinase is recruited to the site of DNA double-strand breaks and subsequently becomes activated, resulting in phosphorylation of multiple substrates, including its main effector kinase Chk2. Single-stranded DNA breaks (SSBs) are, on the other hand, sensed primarily by replication protein A (RPA) protein, which in turn recruits ATM and rad3-related kinase (ATR) through its binding partner, ATRIP. This recruitment activates ATR-promoted phosphorylations, including its main effector kinase, Chk1 [17,18]. ATR is also involved in sensing so-called replication stress (RS). RS is yet another culprit identified as playing a prominent role in driving genomic instability and tumorigenesis [19]. Interestingly, targeting the ATR-Chk1 signaling cascade is being exploited as an attractive approach in cancer treatment [20,21]. Notably, ATR kinase is among the suggested targets of YM-155 [22], as will be discussed further. The rationale behind ATR’s inhibiting anticancer strategy is that such treatment should favor stalled replication forks to collapse, which leads to an increased generation of otherwise deleterious DSBs [21,23]. Another crucial early event in the DSB response is the phosphorylation of histone variant H2AX on a serine 139 by ATM and ATR. When phosphorylated, H2AX is then termed as γH2AX. After irradiation, replications stress, or DNA damaging chemicals, H2AX is rapidly phosphorylated in chromatin areas surrounding DSBs and serves as an anchor for various effector DNA repair proteins. Such regions can be detected by immunofluorescence microscopy as typical γH2AX foci, which can be quantified and thus used as a well-known marker for DSBs [24].

The cells employ two major DNA repair pathways for the repair of DNA double-strand breaks. This is classical-non-homologous end-joining (C-NHEJ) and homologous recombination-mediated repair (HR). C-NHEJ was considered an error-prone DNA repair pathway for a long time, considering DNA break ends are processed by end-cleavage and then directly ligated, resulting in an inevitable loss of bases. However, recent discoveries have shown that C-NHEJ is not fundamentally inaccurate but is rather flexible regarding its inexact ends. Instead, the structure of the DNA ends seems to be the real cause of the end-joining quality [25]. There is also a hypothesis that C-NHEJ proteins form a complex with RNA polymerase II and that the nascent RNA could provide a template for reconstructing the missing sequences, which allows error-free DSB repair of the transcribed genome [26].

In contrast, HR is deemed error-free because of a homologous template, namely sister chromatid, which allows complete replacement of the damaged area with proper DNA sequence. The need for sister chromatid for the HR also means that the process is possible only in the S and G2 phases of the cell cycle [27]. Upon DNA damage, the cell also activates highly coordinated signaling pathways, known as DNA damage checkpoints, responsible for delaying or arresting cell cycle progression. The checkpoints’ role is mainly to provide DNA repair time by minimizing interference with DNA replication, DNA translation, and mitosis [28].

## 3. YM-155 Effects on DNA Integrity

### 3.1. Radio- and Chemosensitizing Effects of YM-155 Are Survivin-Independent

Treatment of ovarian cancer (OVCa) is associated with several obstacles, including the development of chemoresistance of tumors to therapy, which means a poor prognosis for the patient [17]. One of the players of such acquired chemoresistance in many human cancers is survivin [5,29]. Many human cancers overexpress survivin, including OVCa, where it can be detected in as many as 74% of cases, and it is also associated with advanced clinical stages [30]. Thus, survivin’s inhibition would represent a tempting approach to combat the chemo/radioresistance cancer types. For this reason, further research was focused on reported survivin inhibitor YM-155. One of the conclusions was that transcription factor ILF3/NF110, which binds to the promoter of survivin, is, in fact, the molecular target of YM-155 [31]. However, in an attempt to sensitize ovarian cancer (OVCar) cells to cisplatin using YM-155, unexpected results were reported. The expression of survivin did not affect the sensitivity of the cells to YM-155. Moreover, survivin knockdown did not affect the DNA damage response induced by cisplatin. These results suggest that survivin cannot be the only target of YM-155, and the observed sensitizing effect should be survivin-independent [32]. The same surprising survivin expression-independent effect of YM-155 was also reported for p53-deficient T-acute lymphoblastic leukemia (ALL) [33] and renal cell carcinoma [34]. Another study evaluating the radiosensitization effects of YM-155 on esophageal squamous cell carcinoma reported that the treatment abrogated the radiation-induced G2/M arrest [35]. Such a shortening of the G2 checkpoint is responsible for the decreased repair of radiation-induced damage [36] and might explain the radiosensitizing effect. Interestingly, similar irradiation-induced G2/M checkpoint abrogation, causing the radiosensitization effect, was reported for the inhibitors of the ATR-Chk1 pathway [37]. Indeed, ATR kinase is among the newly identified targets of YM-155 [22].

### 3.2. YM-155 Treatment Causes DNA Damage in Cells

Another phenotype reported for YM-155 treatment involves cell cycle arrest irrespective of the cell cycle phase [37]. DNA damage might well explain such perturbation of the cell cycle. Indeed, activation of proteins and pathways involved in DNA damage response, such as ATR, ATM, Chk1 CHk2, and p53, and increased mRNA levels of XRCC5 protein were reported for the YM-155 treatment [38]. Necessary complementary confirmation that YM-155 indeed damages DNA was achieved through the employment of single-cell gel electrophoresis assay (known as a comet assay), which confirmed the induction of double-strand breaks (DSBs). Importantly, survivin silencing did not increase DSBs measured by the comet assay [38]. The research mentioned above also supports the surviving-independent molecular mechanism of YM-155 action. The authors identified YM-155 in a screen for small molecules that could improve the killing of NSCLC by volasertib, a new generation of polo-like kinase 1-PLK1 inhibitors [39]. PLK1 kinase is a protooncogene that drives the cell cycle from the G2 to the M phase [40]. Volasertib, combined with YM-155, displays a synergic effect at nanomolar concentrations resulting in programmed cell death. The same combination also overcame the adaptation of cells to PLK1 inhibition. Importantly, all these effects were achieved at concentrations lower than those needed for a significant survivin downregulation [38].

On the other hand, there is at least one study with contrary survivin-related results. The authors reported that targeting of survivin by siRNA caused DNA damage in MDA-MB-231 and MCF7 cell lines and led to similar cellular responses as observed after the YM-155 treatment. They even suggested a mechanistic explanation. According to the authors, YM-155-mediated survivin suppression induces autophagy-dependent DNA damage, which results in autophagic cell death in various subtypes of breast cancer [41].

### 3.3. Reported YM-155 Toxicity and Cell Cycle Arrest Is p53-Independent

An interesting observation, reporting the preferential cytotoxicity of the BCR-ABL1 oncogene harboring (Ph+ALL) subtype of ALL cancer, showed that the S phase cells were significantly more sensitive to YM-155, suggesting a mechanistic link with the DNA replication. Simultaneously, the Ph+ALL cell line showed only a minimal increase in the p53 phosphorylation after YM-155 [42]. Although the phosphorylation of p53 was reported for YM-155 treatment by others [43], the p53 redundancy for its toxic effect was supported by experiments with mutant p53 cell lines, which confirmed unchanged sensitivity [42]. The context of p53 is also relevant to the reported preferential cytotoxicity of YM-155 against cancer-initiating v-Src oncogene-transformed mammary cells. In this study, early markers of DSBs and initiators of DNA repair were analyzed, pointing to the activation of the ATM-Chk2 pathway. The YM-155 treated cells also slowed down the cell cycle progression and became accumulated preferentially in the S phase. The cell death which followed was dependent on autophagy and NF-κB and entirely independent of p53 status [44].

Autophagy-mediated cell death is an exciting feature of YM155 treatment, as apoptosis is considered the primary cell death program triggered by chemotherapy [45]. However, caspase inhibition did not rescue cancer cells from cell death, which suggests that apoptosis is not an executive cell death pathway triggered by YM155 treatment. The involvement of necroptosis was also ruled out using RIP1 inhibitor Necrostatin-1, which could not prevent YM155-induced cell death. Only blocking the autophagy process with 3-MA or chloroquine potently prevented cell death upon YM155 treatment [44]. The NF-kB pathway and autophagy are essential processes for maintaining cellular homeostasis and play a role in tumorigenesis and cancer treatment resistance. Autophagy is a self-degradative process with tumor-suppressive activity in early oncogenesis, but it can contribute to treatment resistance in later cancer development stages [46]. NF-kB pathway activation plays an essential role in cellular stress responses and is associated with resistance to cancer therapies [47]. It is important to elucidate how novel cancer compounds such as YM155 execute their effects since the delicate interplay between the homeostatic pathways and the apoptotic executive process will ultimately dictate the treatment outcome.

### 3.4. DNA Damage as the Primary Mode of Action of YM-155

Since the beginning of YM-155 research, the most provocative question concerning its cytotoxicity was whether the reported DNA damage is the cause or the consequence of other underlying processes, mainly related to the survivin inhibition. The reasoning was that survivin enhances radiation resistance in glioblastoma cells independently on its antiapoptotic function [11], suggesting its possible role in the DNA damage process. It was observed that irradiation causes survivin translocation from the cytoplasm to the nucleus in radioresistant glioblastoma cells, and this effect was followed by enhanced DSB repair capability [11]. An additional study also supported the hypothesis of survivin’s involvement in DSB repair. The authors likewise observed survivin’s nuclear accumulation following irradiation and revealed an association of survivin with several DSB repair proteins, including Ku70, γH2AX, and DNA-PKcs in nuclear extracts [12].

The initial observation that YM-155 has a radiosensitizing effect in NSCLC led to the investigation of its impact on DSBs repair by measuring γH2AX foci formation and persistence. In the presence of YM-155, γH2AX persisted longer, suggesting DSB’s repair interference. As YM-155 was known to downregulate survivin, delayed repair of radiation-induced DSBs was initially linked to survivin’s potential role in this process [10]. A similar conclusion about the potential role of survivin in DSB repair came from another study where YM-155 was used as a sensitizer to platinum-based compounds (cisplatin and carboplatin). Here, the authors reported a significant delay in the platinum compound-induced DNA damage repair [48]. The first evidence that YM-155 might damage DNA independently of survivin came with the observation that YM-155 is a DNA damage inducer even at doses lower than those needed for survivin suppression [13]. Interestingly, this research article evaluated one more structurally related drug, naphthoquinone imidazolium NSC80467 [49]. Both compounds proved to be highly cytotoxic, and treatment with both drugs resulted in survivin suppression. Both drugs also displayed effects reminiscent of DNA damaging agents, which initially inspired the hypothesis that survivin suppression might be responsible for the phenotype. The authors next tested these drugs’ ability to inhibit DNA, RNA, and protein synthesis, concluding that DNA synthesis was preferentially impaired compared to protein and RNA synthesis [13].

Additionally, both compounds were shown to cause rapid phosphorylation of histone H2AX and the transcriptional repressor KAP1 at nanomolar concentrations. However, surprisingly, survivin protein levels were decreased only at much higher doses, altogether proving the observed phenotype is most likely survivin independent [13]. This surprising discovery reclassifies YM-155 as a DNA damaging agent instead of a specific survivin inhibitor. After these game-changing initial observations, researchers’ opinions on the YM-155 mechanism of action remain somehow divided between a “DNA damaging agent” vs. a “survivin suppressant”.

### 3.5. DNA Damage Pathways Affected by YM-155

Many reports pointed out that YM-155 treatment leads to elevated DNA damage. Since the genome is under constant diverse attacks from free radicals, replication errors, and other DNA damaging factors, one explanation could be a potential interference with some of the DNA damage repair pathways. As already mentioned, mammalian cells rely mainly on two main DSB DNA repair pathways, involving homologous recombination promoted repair (HR) and non-homologous end-joining of broken DNA ends (NHEJ) [50]. The research study concerning radiosensitizing NSCLC by YM-155 revealed that, upon irradiation, survivin quickly accumulates in the nucleus and interacts with DNA-PKcs and KU, which are the known essential factors of NHEJ. Moreover, after the irradiation, YM-155 treatment also decreased the activating autophosphorylation of DNA-PKcs at S2056 [51]. Thus, NHEJ might represent a relevant target of YM-155, explaining the accumulation of DNA breaks after YM-155. However, other studies are focused on HR instead. For example, in breast cancer cells, survivin depletion decreases the transcription of genes involved in HR, namely EME1, BLM, EXO1, BRCA1, BRCA2, and Rad51, of which Rad51 recombinase represents the most significant factor required for HR [52]. A decreased Rad51 protein level was also reported in a study involving Bsl-XL silenced cells, where YM-155 treatment had a potent effect accompanied by increased accumulation of γH2AX and abrogated DSB repair [53]. The interference of YM-155 with the HR-mediated DNA repair was also reported for glioblastoma cells due to reduced levels of Rad51 and BRCA1 (another essential HR factor). This study was also accompanied by a quantitative assay based on an HR reporter revealing approximately 40% reduction in HR promoted DNA repair efficacy after YM-155 treatment [54]. In this study, the authors have also suggested another novel cause of the YM-155 radiosensitizing effect in glioblastoma cells involving cellular invasion inhibition. This is of high significance considering radiation therapy is a standard of care therapy for glioblastoma. Unfortunately, radiation can paradoxically contribute to tumor progression by promoting migration and invasion of irradiated cancer cells [55]. This effect is facilitated by radiation therapy-induced epithelial-mesenchymal transition (EMT) [56]. According to the authors, YM155 treatment can revert the EMT process in glioma cells, which prevents the radiation-induced invasion, most likely via the inhibition of STAT3 [54]. STAT3 is a transcription factor with an essential role in EMT, and it is known that its inhibition leads to the reversal of the EMT process in cancer cells [57]. Reported EMT changes after YM155 were attained by inhibition of STAT3 phosphorylation, and this process was surviving-independent [54]. Importantly, due to the proven role of EMT in cancer progression, STAT3 inhibitors represent yet another group of promising candidates for cancer treatment in combination with radiotherapy [58].

Inhibition of RAD51 foci formation and prolongation of γH2AX signal after gamma irradiation was also reported for YM-155 treated esophageal squamous cell carcinoma [35]. However, in this study, the authors attributed the enhanced radiation effect to survivin inhibition [35].

### 3.6. Other Possible Molecular Mechanisms behind the DNA Damage Caused by YM-155

Apart from the interference with DNA damage repair pathways, there are other potential explanations for the DNA damaging properties of YM-155. For example, the global gene-disruption assay mapping the genetic background responsible for the sensitivity to YM-155 revealed that not the survivin levels but rather the uncharacterized solute carrier SLC35F2, which enables the transport of YM-155, proved to be the real sensitivity promoter. Cells overexpressing SLC35F2 also showed a very high load of DNA damage. The authors explored the potential molecular mechanism more in-depth and concluded that YM-155 exhibits characteristics shared for DNA intercalators. The authors employed the EdU incorporation assay for measuring the pace of replication, and they showed that YM-155 inhibits DNA replication efficiently, which is a feature typical for intercalating agents [59]. This research provided critical mechanistic evidence that the primary mode of YM-155 action could have a direct effect on DNA. Another interesting observation came a few years later, claiming that YM-155 inhibits topoisomerase function. At the same time, this study questioned the reported intercalating properties of YM-155 using viscosity, circular dichroism, and spectroscopy assays. Instead, YM-155 behaves as an inhibitor of Top 2α decatenation and Top-1-mediated cleavage of DNA, which ultimately leads to DNA damage in replicating cells. Moreover, this study confirmed that DNA repair is defective in YM-155 treated cells, namely HR, which might explain the drug’s relatively high potency [60]. The DNA topoisomerases are fundamental for maintaining DNA topology during DNA transcription and replication by relaxing positive or negatively supercoiled DNA [61]. Since they play such a pivotal role in DNA integrity, they become a desirable target in cancer research and drug development [62]. In a clinical setting, topoisomerase inhibitors that act as topoisomerase poisons are successfully used. Unfortunately, they also pose adverse side effects, such as developing secondary malignities. The development of anticancer drugs that target only the isoform-specific human topoisomerase II may represent a safer alternative [63]. Reported inhibition of topoisomerase by YM-155 might give a new, improved formulation of inhibition, potentially overcoming current clinical setbacks. It would also be desirable to uncover combination regimens with other cancer therapeutic drugs, known to be efficient in combination with topoisomerase inhibitors.

Another critical research study on the effects of YM-155 used the whole transcriptome analysis of MDA-MB-231 cells. Over 2000 differentially deregulated transcripts were identified by this approach, overshadowing the loss of survivin’s mRNA. The most affected transcripts included tumor suppressors, DNA replication, cell cycle progression, and DNA damage response factors. In regard to DNA damage, the ataxia-telangiectasia mutated (ATM), Fanconi anemia E3 monoubiquitin ligase core complexes (FANC transcripts—A/B/E/F/G/M), FANC2, FANCI, BRCA1, BRCA2, RAD51, PALB2, and ATR (ATM- and Rad3-related) genes were among the most affected. This research declares that the effect of YM-155 is most likely prompted by affecting the DNA repair machinery [22].

Yet another proposed molecular mechanism standing behind the YM-155-induced DNA damage includes oxidative damage—more precisely, the oxygen-independent redox-activated oxidative DNA damage. In [64], the authors tried to determine the exact molecular mechanism of how YM-155 causes DNA damage. By using cell-based assays, they demonstrated that YM-155 induces considerable DNA cleavage and generation of reactive oxygen species. Although YM-155 is a quinone, it causes DNA damage differently from common quinones [65]. They further show that YM-155 cleaves DNA in the presence of catalase and under hypoxic conditions, which implies that hydrogen peroxide and oxygen are not crucial for this process. Altogether, they suggest that YM155 can cause oxidative DNA cleavage upon two-electron reductive activation [64].

As mentioned before, YM-155 showed promising results in various preclinical studies, which led to multiple clinical studies but with less favorable outcomes [7]. Authors in the next study [66] asked the essential question of whether this could be due to the development of adaptive resistance to YM-155 in cancer patients [67]. The argument was that during phase I clinical studies involving patients with advanced stages of solid tumors that no longer responded to standard therapies, the patients were subjected to up to 168 cycles of YM-155 administration [8,68]. Given the relatively high number of YM-155 cycle administrations, one could propose that adaptive resistance might develop. To test this hypothesis, the authors developed a YM-155-resistant estrogen receptor-positive MCF-7 cell line. The induction of YM-155 resistance in MCF-cells supported the oxidative stress-mediated DNA damage theory. The resistant MCF-7 breast cancer cells exhibited responses common for adaptation to persistent DNA damage induced by oxidation. Additionally, the hypothesis was supported by decreasing the antioxidant glutathione levels, which restored the YM-155 sensitivity [66]. It is worth mentioning that the study also shows that chronic exposure to YM-155 downregulates survivin with the same ability in the drug-resistant and the drug naïve cells, yet again confirming that the antitumor effect of YM-155 cannot be exclusively attributed to survivin suppression [66].

## 4. Conclusions

This review article gives an overview of more than a decade of research on YM-155 and its molecular mechanism of action. The initially proposed but somewhat misleading target, survivin, and further struggle to find a genuine molecular target of YM155 are not unique concerning cancer research. A similar good example of a compound with a similar molecular target history is the alcohol-abuse drug Antabuse (Disulfiram, DSF), a hot candidate for repurposing as an anticancer drug. Although DSF is known for its inhibition of aldehyde dehydrogenase in vivo, its molecular mode of action in targeting cancer suggests the involvement of entirely different pathways such as protein turnover [69] and DNA damage [70]. The historically established link between disulfiram and ALDH inhibition even misled numerous researchers in using this compound as a direct ALDH inhibitor in multiple experiments, completely ignoring the fact that in vivo metabolic processing of DSF is necessary to obtain ALDH inhibitor [71]. Thus, clinical and basic research can be severely affected without the knowledge of the studied compounds’ precise mechanisms of action.

**Figure 1:**
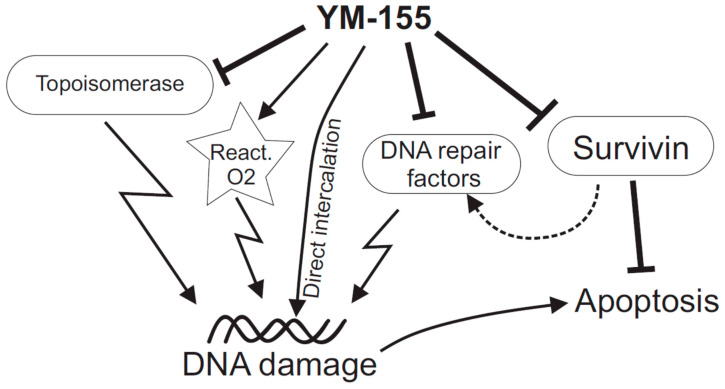
Scheme summarizing some of the suggested mechanisms of action of YM-155 on cancer cells. Apart from the primary target antiapoptotic factor, survivin, there is increasing evidence that YM-155 directly or indirectly damages DNA. The latter scenario may involve direct intercalation into DNA, inhibition of topoisomerases Top 2α and Top-1, various alterations of DNA repair factors (which might also be Survivin-mediated), and emission of reactive oxygen species.

In light of the reports evaluated in this review, it can be concluded that YM-155’s effect on cancer cells preferentially involves DNA damage and DNA repair processes. Whether YM-155 is the intercalating agent, topoisomerases inhibitor, repair pathways disruptor, reactive radical producer, or all of these together (see Figure 1) is still elusive. The historically proposed primary target, survivin, seems to be not relevant or somewhat secondary. From the evaluated reports, it is also clear that YM-155 also fulfills a feature of a multitarget drug. On the one hand, this makes the research of molecular mechanisms rather complicated, explaining numerous suggested mechanisms and even different or contrary conclusions. On the other hand, such a multitarget nature of YM-155 provides an excellent chance to be genuinely efficient against multiple cancer types while minimizing the illness’ adaptation capability.
